# *In vitro* study of *ATP1A3* p.Ala275Pro mutant causing alternating hemiplegia of childhood and rapid-onset dystonia-parkinsonism

**DOI:** 10.3389/fnins.2024.1415576

**Published:** 2024-07-31

**Authors:** Dan-dan Ruan, Jing Zou, Li-sheng Liao, Ming-dong Ji, Ruo-li Wang, Jian-hui Zhang, Li Zhang, Mei-zhu Gao, Qian Chen, Hong-ping Yu, Wen Wei, Yun-fei Li, Hong Li, Fan Lin, Jie-wei Luo, Xin-fu Lin

**Affiliations:** ^1^Shengli Clinical Medical College of Fujian Medical University, Fuzhou, China; ^2^Department of Hematology, Fujian Provincial Hospital, Fuzhou, China; ^3^Department of Emergency, Fujian Provincial Hospital, Fuzhou, China; ^4^Department of Nephrology, Fujian Provincial Hospital, Fuzhou, China; ^5^Department of Rehabilitation Medicine, Ganzhou Municipal Hospital, Ganzhou, China; ^6^Department of Neurology, Fujian Provincial Hospital, Fuzhou, China; ^7^Department of Geriatric Medicine, Fujian Provincial Center for Geriatrics, Fujian Provincial Hospital, Fuzhou, China; ^8^Pediatrics Department, Fujian Provincial Hospital, Fuzhou, China

**Keywords:** *ATP1A3*, Na^+^/K^+^-ATPase, alternating hemiplegia of childhood, rapid-onset dystonia-parkinsonism, zebrafish

## Abstract

**Introduction:**

We previously reported that ATP1A3 c.823G>C (p.Ala275Pro) mutant causes varying phenotypes of alternative hemiplegia of childhood and rapid-onset dystonia-parkinsonism in the same family. This study aims to investigate the function of *ATP1A3* c.823G>C (p.Ala275Pro) mutant at the cellular and zebrafish models.

**Methods:**

ATP1A3 wild-type and mutant Hela cell lines were constructed, and ATP1A3 mRNA expression, *ATP1A3* protein expression and localization, and Na^+^-K^+^-ATPase activity in each group of cells were detected. Additionally, we also constructed zebrafish models with *ATP1A3* wild-type overexpression (WT) and p.Ala275Pro mutant overexpression (MUT). Subsequently, we detected the mRNA expression of dopamine signaling pathway-associated genes, Parkinson’s disease-associated genes, and apoptosisassociated genes in each group of zebrafish, and observed the growth, development, and movement behavior of zebrafish.

**Results:**

Cells carrying the p.Ala275Pro mutation exhibited lower levels of *ATP1A3* mRNA, reduced *ATP1A3* protein expression, and decreased Na^+^-K^+^-ATPase activity compared to wild-type cells. Immunofluorescence analysis revealed that *ATP1A3* was primarily localized in the cytoplasm, but there was no significant difference in *ATP1A3* protein localization before and after the mutation. In the zebrafish model, both WT and MUT groups showed lower brain and body length, dopamine neuron fluorescence intensity, escape ability, swimming distance, and average swimming speed compared to the control group. Moreover, overexpression of both wild-type and mutant *ATP1A3* led to abnormal mRNA expression of genes associated with the dopamine signaling pathway and Parkinson’s disease in zebrafish, and significantly upregulated transcription levels of *bad* and *caspase-3* in the apoptosis signaling pathway, while reducing the transcriptional level of *bcl-2* and the *bcl-2/bax* ratio.

**Conclusion:**

This study reveals that the p.Ala275Pro mutant decreases *ATP1A3* protein expression and Na^+^/K^+^-ATPase activity. Abnormal expression of either wild-type or mutant *ATP1A3* genes impairs growth, development, and movement behavior in zebrafish.

## Introduction

Na^+^/K^+^-ATPase is a crucial transmembrane ion pump that uses the energy from hydrolyzed adenosine 5′-triphosphate to actively transport three Na^+^ ions out of the cell in exchange for two extracellular K^+^ ions transported into the cell (Miyatake et al., 2021). The electrochemical ion gradient established and maintained by Na^+^/K^+^-ATPase at the plasma membrane is essential for restoring resting membrane potential, supporting electrical activity of excitable cells, regulating ion transport, and driving neurotransmitter signal transduction (Holm et al., 2016). Meanwhile, alterations in intracellular Na^+^ and K^+^ homeostasis play a regulatory role in the initial signal transduction and execution phases of apoptosis (Panayiotidis et al., 2006). Na^+^/K^+^-ATPases are primarily composed of the α and β subunits. In humans, there are four different isoforms of the α subunit (α1–α4), each with distinct tissue expression patterns. In the central nervous system (CNS), the α1 isoform exhibits widespread expression, α2 isoform is predominantly expressed in astrocytes and glia cells, and the high expression of α3 isoform in the brain is mainly localized in neuronal projections (Clausen et al., 2017). Pathogenic mutants in the *ATP1A3* gene, encoding the α3 isoform, can lead to *ATP1A3*-related neurological disorders such as alternating hemiplegia of childhood (AHC), rapid-onset dystonia-parkinsonism (RDP), and cerebellar ataxia, areflexia, pes cavus, optic atrophy, and sensorineural hearing loss (CAPOS) syndrome (Sharawat et al., 2020). These three different clinical phenotypes were initially thought to be independent, but with the emergence of overlapping phenotypes and an increase in the number of atypical cases, *ATP1A3*-related disorders sometimes seem to be part of a continuous clinical spectrum (Duat-Rodriguez et al., 2021; Ng, 2021). Interestingly, the severity of the disease varies widely, and there is a broad range in both the age of onset and the progression of the disease (Vezyroglou et al., 2022). Dysfunction of *ATP1A3* that occurs in childhood may not show corresponding effects until adulthood (Zou et al., 2023).

Zebrafish have two *ATP1A3* orthologues, *atp1a3a* and *atp1a3b*, which exhibit high homology to human *ATP1A3* (Ng et al., 2021). Zebrafish are characterized by their small size, ease of reproduction, and transparency during embryonic and early larval stages. Additionally, the overall structure of many brain regions in zebrafish (such as the retina, olfactory bulb, cerebellum, and spinal cord) is similar to that of humans. Therefore, zebrafish serve as a favorable model for studying neurological disorders (Friedrich et al., 2010). *ATP1A3* isoforms play a crucial role in restoring electrochemical gradients in neuronal activity. However, the molecular basis of the many different neural syndromes caused by *ATP1A3* mutations is currently unclear. In previous clinical work, we observed that the *ATP1A3* gene c.823G > C (p.Ala275Pro) mutant resulted in different clinical phenotypes (AHC and RDP) in the same family (Case presentation is available in the Supplementary materials). Genetic-phenotypic correlation analysis of the family suggested that this mutant might be a pathogenic mutation (Wei et al., 2022). Here, we constructed overexpression plasmids for *ATP1A3* wild-type and *ATP1A3* c.823G > C (p.Ala275Pro) mutant and analyzed the effects of c.823G > C mutation on *ATP1A3* protein expression, localization, and Na^+^/K^+^-ATPase activity at the cellular level. Additionally, we constructed zebrafish models with *ATP1A3* gene mutation. By observing the growth, development, and motor behavior of zebrafish, and analyzing the mRNA expression of dopamine signaling pathway-associated genes, Parkinson’s disease (PD)-associated genes, and apoptosis-associated genes after *ATP1A3* mutation, we further identified the function of the *ATP1A3* p.Ala275Pro mutant and explored the influence of this mutant on the zebrafish nervous system.

## Methods

### Zebrafish

In this study, we used wild-type AB strain zebrafish and transgenic zebrafish of the *Tg (vmat2:GFP)* line, which has green fluorescent protein (GFP)-labeled in dopamine neurons. The male and female zebrafish were raised separately at the standard temperature of 28°C, with a light cycle of 14 h of light and 10 h of darkness per day. They were fed saltwater shrimp twice daily at scheduled times.

### Ethics statement

All breeding processes were strictly implemented in accordance with the standards of the zebrafish experimental manual. All methods were reported in accordance with ARRIVE guidelines. The study was approved by the Ethics Committee of Fujian Provincial Hospital.

### Construction and identification of plasmids

The coding sequence (CDS) of *ATP1A3* (NM_152296.4) and the CDS of the mutation site c.823G > C (p.Ala275Pro) were cloned into pCDH-CMV-MCS-EF1-copGFP-T2A-puro vector, and XbaI/NotI was selected as double restriction enzyme cutting site. The target gene was amplified and verified, and the positive clones were identified by sequencing. The *ATP1A3* gene synthesis and plasmid construction were conducted with support from Wuhan Gene Create Co., Ltd. (Wuhan, China).

### Cell culture and construction of stable cell lines

Hela cells and HEK293T cells were generously provided by Xiamen Lifeint Technology Co., Ltd. Both cell lines were cultured in DMEM complete medium supplemented with 1% penicillin/streptomycin and 10% fetal bovine serum. Cells were incubated in a constant temperature incubator (37°C, 5% CO2). HEK293T cells were evenly spread on a 10 cm cell culture plate containing 15 mL of complete culture medium at a cell amount of 6 × 10^6^ to 7 × 10^6^, and incubated overnight in an incubator containing 5% CO2 at 37°C. The lentiviral packaging experiment can be started after the cells adhered to the wall and grew to about 70–90% density. The empty plasmid pCDH-CMV-MCS-EF1-copGFP-T2A-Puro, overexpression plasmids pCDH-CMV-ATP1A3-EF1-copGFP-T2A-Puro and pCDH-CMV-ATP1A3 mut-EF1-copGFP-T2A-Puro, along with lentivirus packaging plasmid (12 μg lentivirus plasmid +12 μg Lenti-Mix = 24 μg Total, in which Lenti-Mix was made up of pMDLg/pRRE, pVSV-G, pRSV-Rev in a ratio of 5, 3, 2) were added into 5 mL EP tube. Then add 1 mL of 0.25 M CaCl2 and 1 mL of 2 x HBS, mix well, and allowed to stand at room temperature for 10 min. Finally, add the Lenti-DNA-Mix transfection system dropwise into the HEK293T cell culture dish to be transfected, mix gently, and place it in a cell culture incubator at 37°C with 5% CO2 for cultivation. After 8–10 h of transfection, the medium was replaced with fresh complete medium and incubated further. Fluorescence observation and photographs were taken 48 h after transfection. After 24 h or 48 h of transfection, the supernatant of cell culture fluid was collected and centrifuged for 10 min at 3000 × g at 4°C. The supernatant was filtered to obtain the lentiviral supernatant. Hela cells were spread on a 10 cm cell culture dish at a density of 4 × 10^5^ to 8 × 10^5^ cells /cm^2^. After the cells completely adhered to the wall, 2 mL of lentiviral supernatant and 10 mL of complete culture medium were added, followed by the addition of polybrene with a final concentration of 10 μg/mL. The lentivirus-containing medium was removed after 24 h of infection. After repeating the infection for 24 h, the cells were replaced with fresh complete medium and the infection was continued until 72–96 h. Observe the cell fluorescence. Stable cell lines were screened by culturing them in complete medium containing puromycin at a final concentration of 0.1 μg/mL.

### Construction of zebrafish model

The vector uses pcDNA3.1, and human wild-type *ATP1A3* and *ATP1A3* c.823G > C mutant sequences carrying SP6 and kozak were connected to Hind III and EcoR I in the multiple cloning sites region of the pcDNA3.1 plasmid by synthetic methods. Following the protocol of the Invitrogen mMESSAGE mMACHINE™ SP6 Transcription Kit, capped mRNA for wild-type and mutant *ATP1A3* was synthesized. Qualified capped mRNA samples were mixed in appropriate concentration ratios to prepare injection samples. These samples were injected into 1-cell stage zebrafish embryos derived from a hybridization between Tg(etvmat2:gfp) strain zebrafish and wild-type zebrafish. Following injection, the zebrafish embryos were incubated in E3 solution at 28.5°C. Experimental groups included: a control group injected with phenol red; wild-type *ATP1A3* overexpression group (WT), injected with WT-*ATP1A3* mRNA; *ATP1A3* c.823G > C mutation overexpression group (MUT), injected with MUT-*ATP1A3* mRNA; and rescue group (WT + MUT), injected with WT-*ATP1A3* mRNA + MUT-*ATP1A3* mRNA.

### Growth, development, and behavior trajectory analysis of zebrafish

When zebrafish developed to 48 h post-fertilization (hpf), the brain size and body length phenotypes of four groups of zebrafish were assessed. Subsequently, at 48 hpf, zebrafish underwent a tail-touch escape experiment after stripping their membranes. Each zebrafish larva was gently touched at least twice on the tail or trunk using the tip of a fine needle on a 10-cm-diameter cell culture dish. If a fry did not move at least three times its own body length after being touched, this was considered a mobility defect. When zebrafish developed to 72 hpf, we photographed the green fluorescence emitted from the zebrafish heads and calculated the fluorescence intensity. This analysis was conducted to assess the development of dopamine neurons in the zebrafish. At 96 hpf, zebrafishes were placed into a 96-well plate, with each zebrafish occupying a separate well. They were then placed in a light incubator and allowed to develop until 120 hpf. At this stage, a 5 min behavior trajectory test was conducted to evaluate swimming behavior. This test included measurements of swimming distance and average swimming speed.

### Quantification of gene expression

The stable cell lines were harvested for RNA extraction followed by reverse transcription. The expression levels of *ATP1A3* mRNA relative to the internal control GAPDH were quantified using SYBR Green qPCR Master Mix (Roche, Switzerland), following the manufacturer’s instructions. Primers were designed using Primer 5.0 software (Table 1). After zebrafish embryos injected with mRNA developed to 24 hpf and 120 hpf, total RNA was extracted from zebrafish tissues using RNAiso Plus (Takara, Japan). RNA samples from each experimental group were reverse transcribed into cDNA. At 24 hpf, ChamQ SYBR qPCR Master Mix (Vazyme, China) was used to quantify the expression of the *ATP1A3* gene in zebrafish, evaluating the effects of *ATP1A3* overexpression. At 120 hpf, the expression levels of the dopamine signaling pathway-associated genes (*th1, th2, mao, dat, drdla, drdlb, drd2a, drd3, drd4a, drd4b*, and *drd5a*), Parkinson’s disease (PD)-associated genes (*dj1, ink1, parkin, lrrk2, polg, gba, sncb, sncga, sncgb*, and *syn2b*), and apoptosis-related genes (*bcl-2, bax, bad, cox4i1, apaf-1, caspase-3, caspase-9*, and *bcl-2/bax*) were measured. The housekeeping gene *elfa* was used as an internal control. Primer design was performed using Primer 5.0 software (Table 2). The mRNA expression levels of each gene were quantified using the 2^−△△Ct^ relative quantification method.

**Table 1:** Primers used in real-time quantitative polymerase chain reaction (cells).

Primer name	Primer sequence (5′-3)
hATP1A3 qRT	F:CTGTCAGAGACAGGGTGCAA
	R:ATTGCTGGTCAGGGTGTAGG
hGAPDH	F:CAAGGTCATCCATGACAACTTTG
	R:GTCCACCACCCTGTTGCTGTAG

**Table 2:** Primers used in real-time quantitative polymerase chain reaction (Zebrafish).

Primer name	Primer sequence (5′-3)
*elfa*	F:CTTCTCAGGCTGACTGTGC
	R:CCGCTAGCATTACCCTCC
*atp1a3*	F:TCATGTAGGGGAGCAGGCAT
	R:CCGAGAGCCTGGATCATTCC
*th1*	F:TCAGAAGTTTGTTGGGAGG
	R:CTGGTTTCAAGATGGTGGA
*th2*	F:GTCTCGCTTGGAGCATCAG
	R:TTAGAAAGGGCATACAACA
*mao*	F:TGCTGAAGAATGGGACAAG
	R:AGAAACCACAAAGCCGAAA
*dat*	F:AGACTCCATCCCTCCCATAGC
	R:CATCATTTACCCAGAAGCCATT
*drd1a*	F:ATTTGGGTCGCATTTGATA
	R:CACCCTGGGAGTCATCTTC
*drd1b*	F:TGGGAAACACGTTGGTCTGT
	R:AACCTACAATCTCCGTGGCG
*drd2a*	F:ATCACAAACCGAAACCCTT
	R:GACCTGGATCTCAAATGCT
*drd3*	F:TGGGCAGTTTATTTGGAAG
	R:GCTGTGGGTGGTGTTGTAT
*drd4a*	F:ACCACCACCAACTACTTCAT
	R:CAGTCCATCACATACGGTCA
*drd4b*	F:TCAAGACGACGACAAACTA
	R:ACATTCAAGGACCAAACAC
*drd5a*	F:CTGCGTCAGCGACACCACCT
	R:GAGAAGCCCTTGCGGAACT
*sncga*	F:GCAGCCGCCAAAACCAAAGA
	R:CCAGCCACAGCAGTATCACT
*sncgb*	F:TCCAGAAGACCACCGACCAG
	R:TTCCATACCACCATGAGAGA
*polg*	F:GCTCTGGTTTTTGATGTGGA
	R:CTGGAGTTTGCGAGGGTTTC
*sncb*	F:CAGCAAGACCAAAGACAGCG
	R:TGGCTTCCTGACCAAACTCC
*lrrk2*	F:TATTCGTTCGGTCTGCTGCT
	R:TGTCCTGGGGGTTTTCTCTC
*parkin*	F:CCATCACCAAAACCACTCAC
	R:GCACCACTCGAACTTACACT
*pink1*	F:GTACCTGGAGGTGTGTGTGC
	R:CTCTGTGAGCGATGTTTTGT
*syn2b*	F:AGTCATCCCATCCTCTCTTG
	R:CTCTGCCTTTGCTTCGTCCT
*bax*	F:GGCTATTTCAACCAGGGTTCC
	R:TGCGAATCACCAATGCTGT
*bad*	F:GCACGCAAGACGTTCAATCA
	R:TTTTTCTGTGCCGACCCACT
*caspase3*	F:TCGCAGGACAGGCATGAAC
	R:GTGATCGTCATGGGCAACTG
*caspase9*	F:TTCTTCAGCGGCACAGGTTA
	R:GTCTGGTTGCCTTGCTCTGTA
*apaf1*	F:TTCCGTCAGCGGTGTAAGGT
	R:TTACAATGCTGCGGGCCTGT
*bcl2*	F:ATGTGCGTGGAAAGCGTCAAC
	R:GAAGGCATCCCAACCTCCATT

### Western blot analysis

Total protein from each group of cells was extracted and quantified using the BCA method to determine protein concentration. Subsequently, protein samples and a protein Marker were loaded into wells on an electrophoresis gel in the desired order. After electrophoresis, the separated proteins were transferred to a polyvinylidene fluoride (PVDF) membrane. Next, the PVDF membrane was blocked with blocking buffer at room temperature for 1 h. The blocked membrane was then incubated overnight at 4°C with a diluted primary antibody solution (Anti-FLAG, 1:2000; Anti-β-Actin, 1:3000). After incubation with primary antibodies, the membrane was washed three times and then incubated at room temperature for 1 h with a diluted horseradish peroxidase-labeled secondary antibody solution. Following another round of washing (five times), the membrane was exposed to an enhanced chemiluminescence reagent, which were subsequently photographed. The intensity of each band was analyzed using Image J software to quantify the gray values.

### Cellular immunofluorescence

After the transfected Hela cells were fixed, permeabilized, and blocked, they were incubated overnight at 4°C with a primary antibody (Anti-FLAG, 1:1000 dilution). The following day, the cells were washed three times with phosphate-buffered saline (PBS). Subsequently, a fluorescent secondary antibody (1:500 dilution) was applied to the cells and incubated at room temperature for 2 h in the dark. After incubation, the cells were washed three times with PBS. After that, the cells were stained with 4′,6-diamidino-2-phenylindole at room temperature for 5 min to visualize the cell nuclei. After staining, the cells were washed twice with PBS. Finally, the cells were mounted with 50% glycerin, and imaged using a laser confocal microscope to observe and capture fluorescence signals from the tagged proteins.

### Na^+^/K^+^-ATPase activity detection

The Na^+^/K^+^-ATPase activity was detected using the Na^+^/K^+^-ATPase Assay Kit. Na^+^/K^+^-ATPase catalyzes the breakdown of ATP to generate ADP and inorganic phosphate. The activity of Na^+^/K^+^-ATPase was quantified by measuring the amount of inorganic phosphate produced. Specifically, 1 unit of enzyme activity is defined as 1 μmoL of inorganic phosphate generated from the breakdown of ATP by Na^+^/K^+^-ATPase per hour per milligram of tissue protein.

### Statistical analysis

Data were statistically analyzed using GraphPad Prism version 6.0 software and SPSS 26.0 software. The measurement data were expressed as means ± SEM. The counting data were expressed as constituent ratio (%). One-way ANOVA was used for comparison among multiple groups, and the LSD test was used for comparison between two groups. *p* < 0.05 was considered statistically significant.

## Results

### Cloning of *ATP1A3* wild-type and p.Ala275Pro mutant

**Figure 1:**
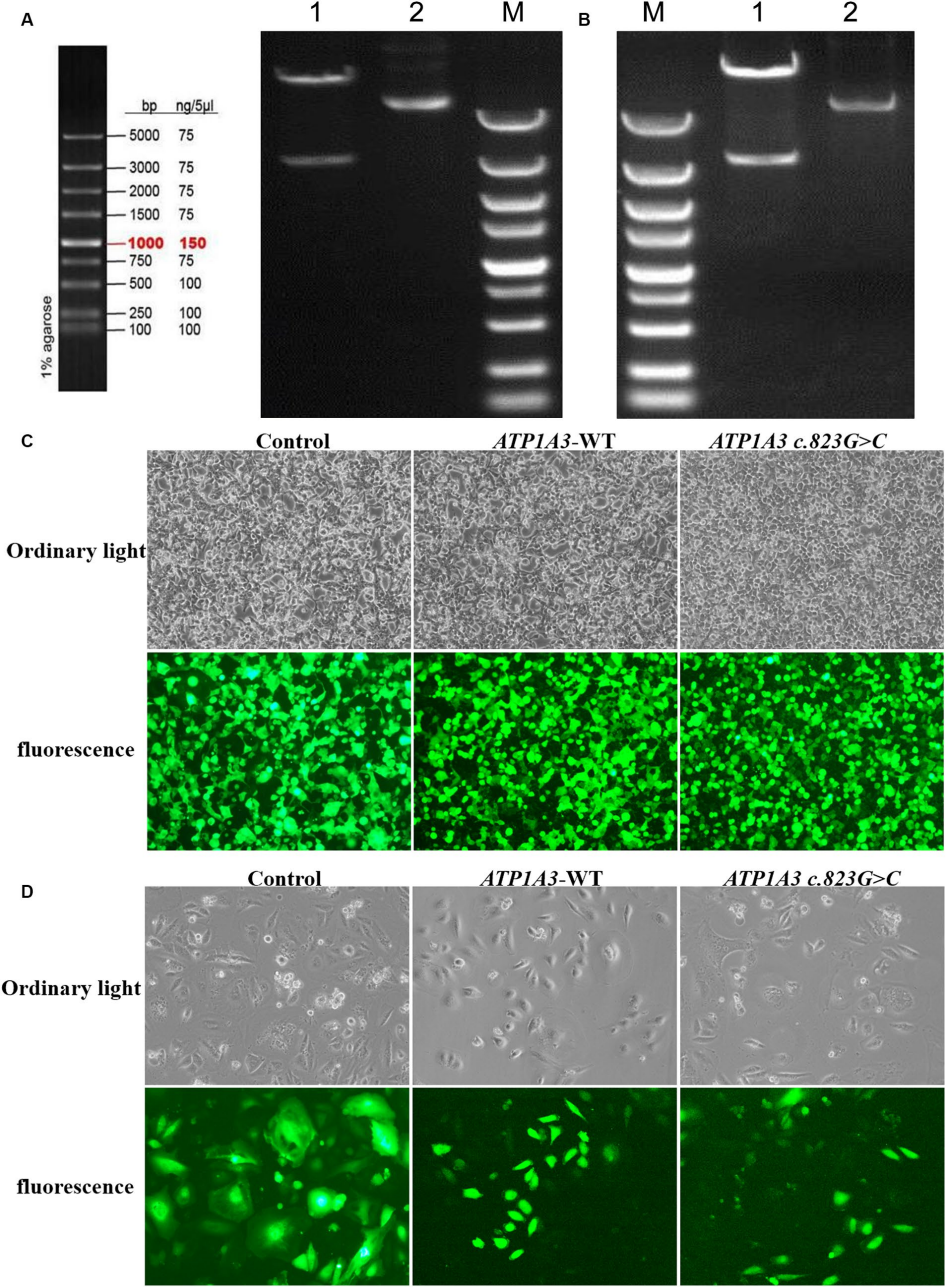
Plasmid construction and cell culture growth. **(A)** The left graph shows the schematic diagram of the marker, and the right graph shows the electropherogram of *ATP1A3* wild type cloned plasmid digested with XbaI/NotI. Lane 1 is plasmid digested by XbaI-NotI, Lane 2 is plasmid DNA, and Lane M is DNA marker. **(B)** Electropherogram of *ATP1A3* (p.Ala275Pro) cloned plasmid digested with XbaI/NotI. Lane 1 is plasmid digested by XbaI-NotI, Lane 2 is plasmid DNA, and Lane M is DNA marker. **(C)** Green fluorescent protein expression after transfection of cells carrying unloaded (control), wild-type (*ATP1A3*-WT), and *ATP1A3* c.823G > C mutant, suggesting that there was no significant difference in transfection efficiency between the groups. **(D)** Cell fluorescence of stable cell lines carrying control, *ATP1A3*-WT, and *ATP1A3* c.823G > C mutant.

After cloning the *ATP1A3* wild-type (*ATP1A3*-WT) and p.Ala275Pro mutant genes, the genes were packaged into lentiviral vectors. The next steps typically involve transforming these vectors into *E. coli* cells and subjecting them to double enzyme digestion (Figures 1A,B). The successfully constructed plasmids were verified by sequencing. The packaged *ATP1A3*-WT and *ATP1A3* c.823G > C plasmids were successfully transfected into HEK293T cells (Figure 1C). After 48 h of transfection, the lentivirus supernatant collected from the transfected HEK293T cells successfully infected Hela cells (Figure 1D).

### Localization of *ATP1A3*-WT and p.Ala275Pro mutant in cells

**Figure 2:**
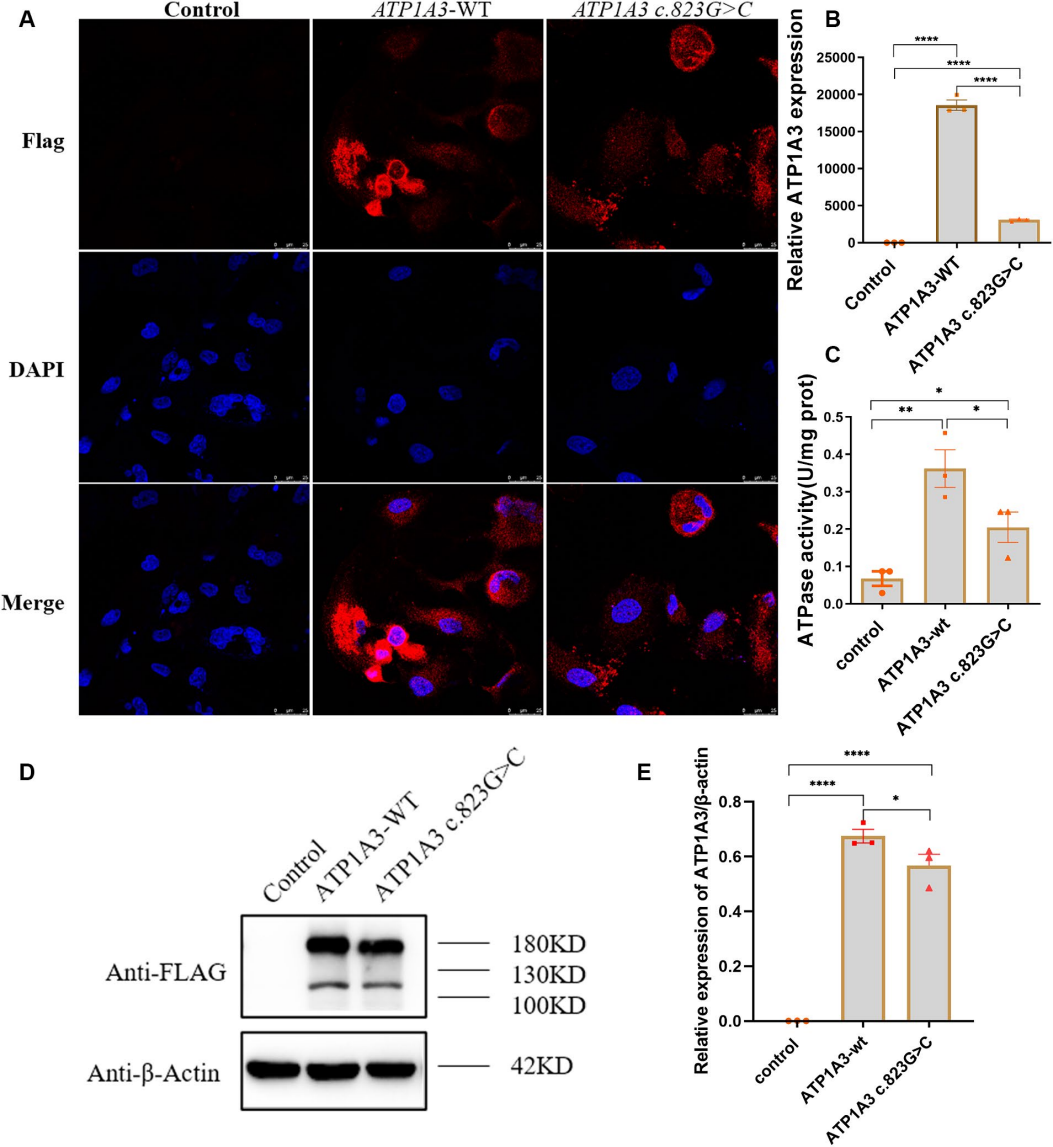
Effect of the c.823G > C mutation on *ATP1A3* gene expression and Na^+^/K^+^-ATPase activity in cells. **(A)** Immunofluorescence detection of *ATP1A3* protein expression and localization in cells carrying the unloaded (control), wild-type (*ATP1A3*-WT), and *ATP1A3* c.823G > C mutants. *ATP1A3* protein was mainly distributed in the cytoplasm, and the expression of *ATP1A3* protein was weaker in cells after the c.823G > C mutation compared with the *ATP1A3*-WT group, but the localization of *ATP1A3* protein after the mutation was not significantly different from that of the *ATP1A3*-WT group. **(B)** RT-qPCR showed that the mRNA level of *ATP1A3* in cells after c.823G > C mutation was significantly lower than that in the *ATP1A3*-WT group. **(C)** The Na^+^/K^+^-ATPase activity was up-regulated in the cells of both *ATP1A3*-WT and *ATP1A3* c.823G > C groups compared to the control group, but the Na^+^/K^+^-ATPase activity was decreased in the cells after the c.823G > C mutation compared to the *ATP1A3*-WT group. **(D)** Using an anti-Flag antibody against Flag-tagged *ATP1A3* protein, the expression of Flag-*ATP1A3* protein in cell lysates of control, *ATP1A3*-WT, and *ATP1A3* c.823G > C groups was detected by western blot (WB). The WB quantification data are shown in **(E)**. Data are presented as mean ± SEM, *N* = 3; NS, not significant, *p < 0.05, **p < 0.01, ***p < 0.001, and ****p < 0.0001.

Immunofluorescence was employed to assess the expression and localization of *ATP1A3* protein in Hela cells. The *ATP1A3* protein was primarily localized in the cytoplasm of the cells. After the c.823G > C mutation, the expression of *ATP1A3* protein in the cells appeared weaker compared to the *ATP1A3*-WT group (Figure 2A). However, there was no significant difference in the localization of *ATP1A3* protein after c.823G > C mutation (Figure 2A).

### Expression of *ATP1A3*-WT and p.Ala275Pro mutant in cells

The expression levels of *ATP1A3*, internal control, and external controls (copGFP), were assessed via RT-qPCR. The findings indicated a substantial reduction in *ATP1A3* mRNA levels in cells after the c.823G > C mutation compared to those in the *ATP1A3*-WT group (Figure 2B). The expression of *ATP1A3* protein in cell lysates was assessed via WB. The findings showed a significant increase in *ATP1A3* protein levels upon overexpression of both wild-type and mutant *ATP1A3* constructs. However, in cells carrying the c.823G > C mutation, *ATP1A3* protein expression was notably reduced compared to the *ATP1A3*-WT group (Figures 2D,E).

### Effect of *ATP1A3* mutation on Na^+^/K^+^-ATPase activity

After overexpressing both wild-type and mutant *ATP1A3*, Na^+^/K^+^-ATPase activity increased in the cells. However, after the c.823G > C mutation, Na^+^/K^+^-ATPase ctivity decreased compared to the *ATP1A3*-WT group (Figure 2C).

### Effect of *ATP1A3* gene on motor behavior, growth, and development of zebrafish

**Figure 3:**
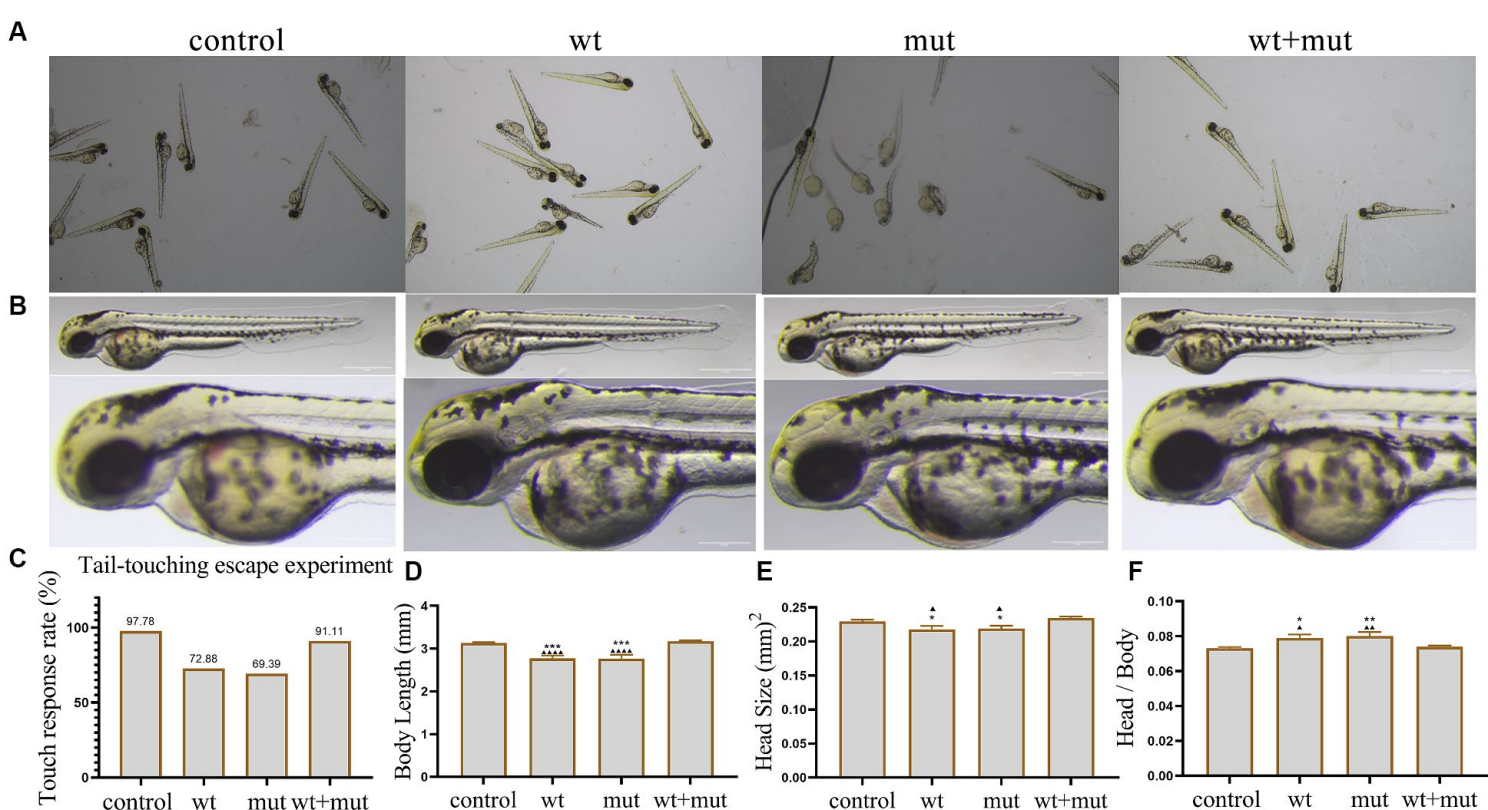
Effects of *ATP1A3* gene on tail-touch escape ability and growth and development of zebrafish. **(A)** Representative images of zebrafish touch-tail escape experiments at 48 hpf in control, wild-type *ATP1A3* overexpression (wt), *ATP1A3* c.823G > C mutation overexpression (mut), and rescue (wt + mut) groups. **(B)** Representative images of the brain and overall morphology of zebrafish in control, wt, mut, and wt + mut groups photographed in white light at 48 hpf. **(C–F)** Statistical graphs of touch-tail escape experiments, body length, brain size, and brain size/body length data for zebrafish in control, wt, mut, and wt + mut groups. ^*^p < 0.05, ^**^p < 0.01, ^***^p < 0.001, and ^****^p < 0.0001 vs. the control group. ^▲^*p* < 0.05, ^▲▲^*p* < 0.01, ^▲▲▲^*p* < 0.001, and ^▲▲▲▲^*p* < 0.0001 vs. the wt + mut group.

**Figure 4:**
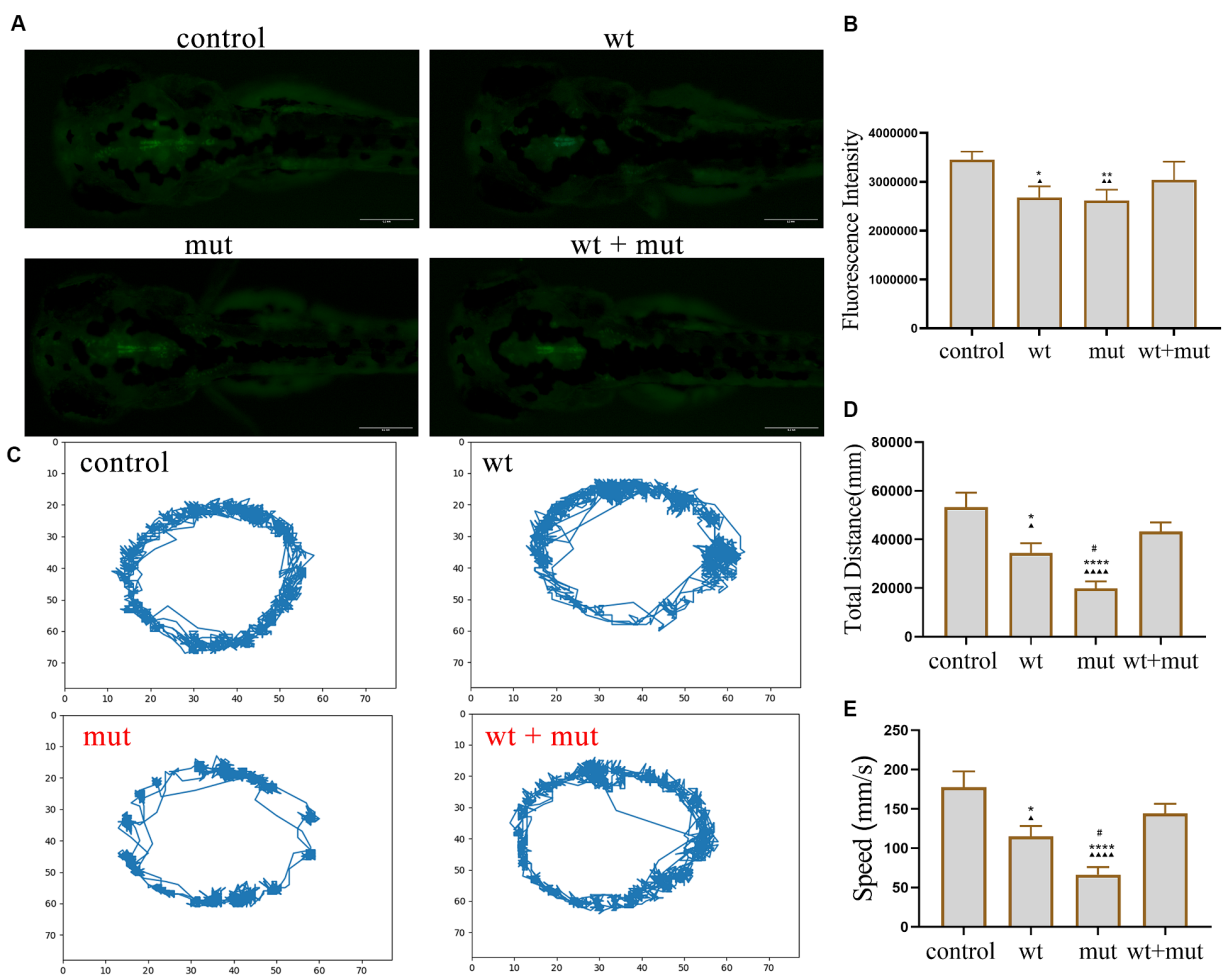
Effects of *ATP1A3* gene on dopamine neuron development and behavioral trajectory of zebrafish. **(A)** Representative graphs of dopamine neuron fluorescence in the brain of zebrafish at 72 hpf in control, wild-type *ATP1A3* overexpression (wt), *ATP1A3* c.823G > C mutation overexpression (mut), and rescue (wt + mut) groups. **(B)** Statistical graphs of the dopamine neuron fluorescence area in the brain of zebrafish in control, wt, mut, and wt + mut groups. **(C)** Representative graphs of the behavioral trajectories of each group of zebrafish as they developed to 120 hpf. **(D,E)** Statistical graphs of the data on the swimming distance and average swimming speed of zebrafish in each group. ^*^p < 0.05, ^**^p < 0.01, ^***^p < 0.001, and ^****^p < 0.0001 vs. the control group. ^▲^*p* < 0.05, ^▲▲^*p* < 0.01, ^▲▲▲^*p* < 0.001, and ^▲▲▲▲^*p* < 0.0001 vs. the wt + mut group. ^#^*p* < 0.05 vs. the wt group.

The results from the tail-touch escape experiments indicated a higher proportion of zebrafish with impaired escape ability in the WT and MUT groups. Notably, the MUT group exhibited the highest proportion of zebrafish with impaired escape ability. In contrast, both the WT + MUT group and the control group showed a lower proportion of zebrafish with impaired escape ability (Figures 3A,C). When zebrafish developed to 48 hpf, the body length and brain size of zebrafish in the WT and MUT groups were observed to be smaller compared to those in the control group. There was no significant difference in body length and brain size between the WT and MUT groups. Conversely, zebrafish in the WT + MUT group exhibited larger body length and brain size than those in the WT and MUT groups (Figures 3B,D,E). In addition, the brain size/length of zebrafish in the WT and MUT groups were higher than those in the control and WT + MUT groups (Figure 3F). When zebrafish developed to 72 hpf, it was shown by statistical analysis of dopamine neuron fluorescence in the zebrafish brain that the dopamine neuron fluorescence intensity of zebrafish in the WT and MUT groups was lower than that of the control and WT + MUT groups, while there was no significant difference between the WT and MUT groups (Figures 4A,B). When zebrafish developed to 120 hpf, analysis of their behavioral trajectories indicated that the swimming distance and average movement speed of zebrafish in the WT and MUT groups were reduced compared to those in the control group. The decrease in both swimming distance and average movement speed was more pronounced in the MUT group specifically. Conversely, zebrafish in the WT + MUT group exhibited higher swimming distance and average movement speed compared to both the WT and MUT groups (Figures 4C–E).

### Effects of *ATP1A3* gene on dopamine signaling pathway-associated genes in zebrafish

**Figure 5:**
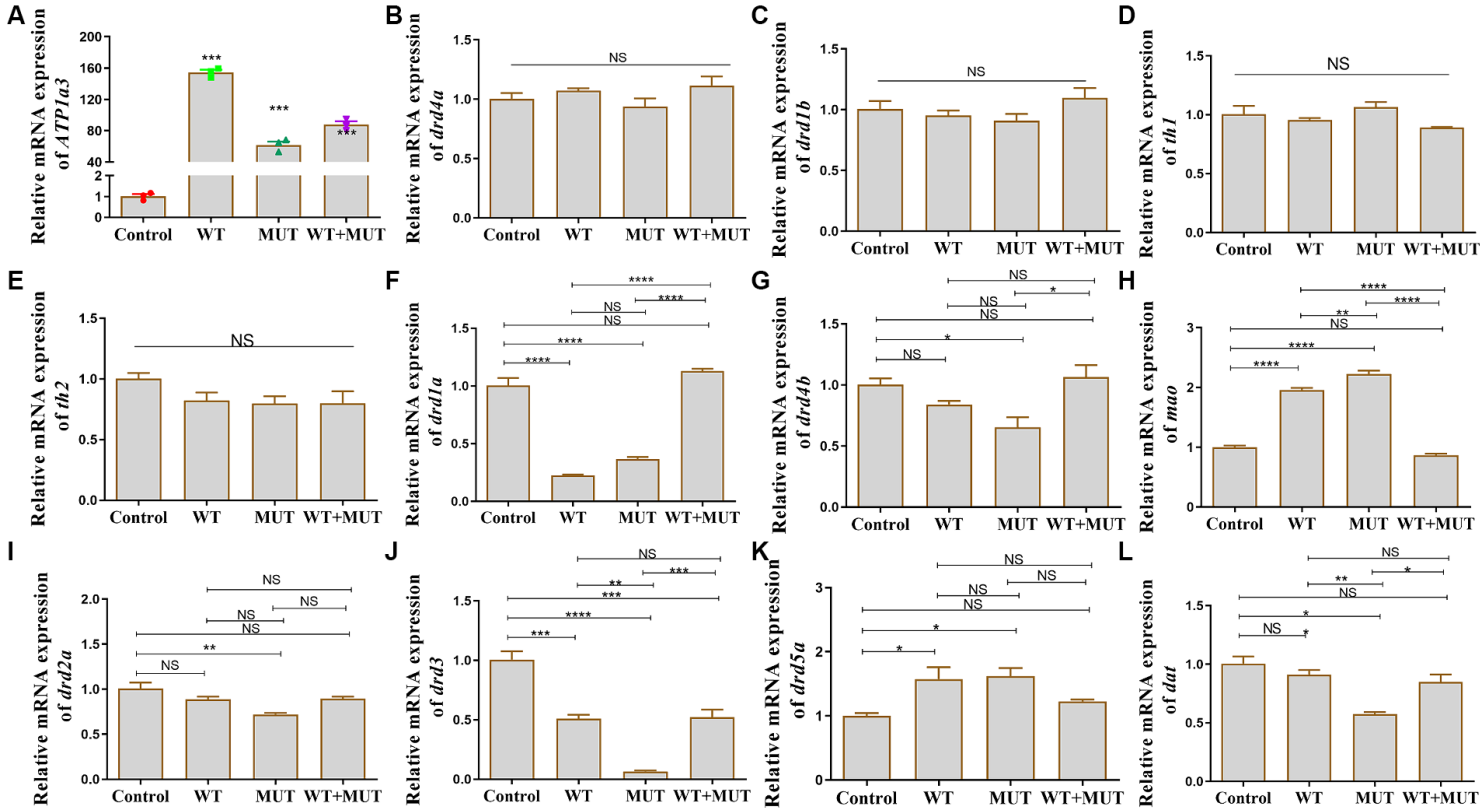
Effect of *ATP1A3* gene on dopamine signaling pathway-associated genes in zebrafish. **(A)** Quantitative analysis of the *ATP1A3* gene in zebrafish from control, wild-type *ATP1A3* overexpression (WT), *ATP1A3* c.823G > C mutation overexpression (MUT), and rescue (WT + MUT) groups when zebrafish development to 24 hpf. The *ATP1A3* expression of the experimental and control groups had a significant difference, suggesting that the overexpression model was successfully constructed and subsequent experiments could be carried out. **(B–L)** Relative mRNA expression of dopamine signaling pathway-associated genes in each group of zebrafish when they developed to 120 hpf. NS, not significant, ^*^p < 0.05, ^**^p < 0.01, ^***^p < 0.001, and ^****^p < 0.0001.

After the overexpression zebrafish models developed to 24 hpf, there was a significant difference in *ATP1A3* mRNA expression between the experimental and control groups (Figure 5A), suggesting that the overexpression model was successfully constructed, allowing for subsequent experiments. Compared to the control group, mRNA levels of *drd1a* and *drd3* were decreased in both the WT and MUT groups, while levels of *mao* and *drd5a* were increased, and the expression of these genes was partially restored in the WT + MUT group (Figures 5F,H,J,K). The mRNA level of *drd3* in the WT + MUT group was lower than that in the control group, but there was no significant difference in *drd1a*, *mao*, and *drd5a* between the WT + MUT group and the control group (Figures 5F,H,J,K). The mRNA levels of *drd4b*, *drd2a*, and *dat* were all decreased in the MUT group compared with the control group, and the expression of *dat* in the MUT group was also lower than that in the WT group; the mRNA levels of *drd4b*, *drd2a*, and *dat* were partially restored in the WT + MUT group, and there was no significant difference in these genes between the WT + MUT group and the control group (Figures 5G,I,L). However, overexpression of wild-type and mutant *ATP1A3* did not significantly affect the mRNA levels of the *drd4a*, *drd1b*, *th1*, and *th2* genes in zebrafish (Figures 5B–E).

### Effects of *ATP1A3* gene on PD-associated genes in zebrafish

**Figure 6:**
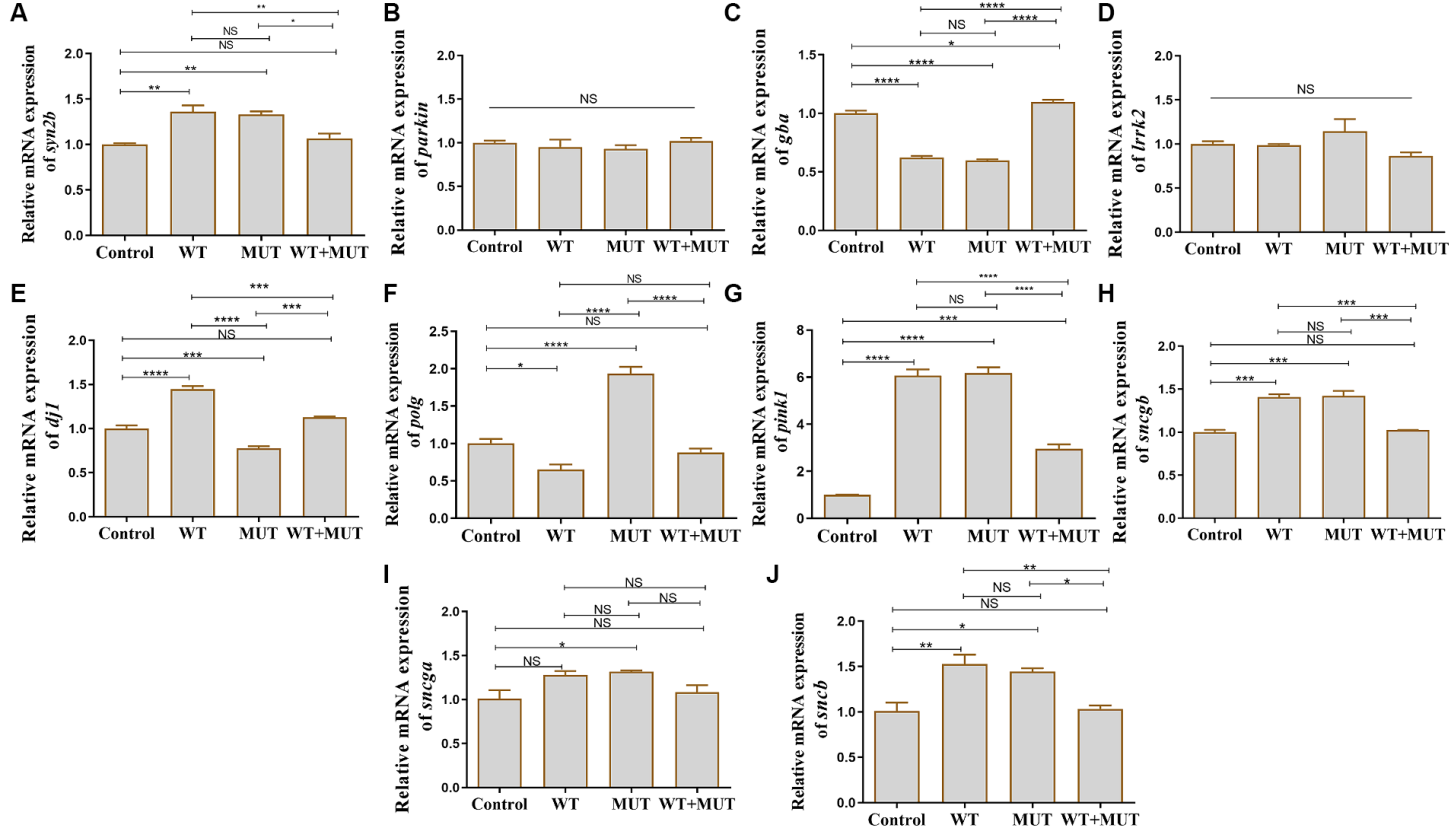
Effects of the *ATP1A3* gene on Parkinson’s disease-associated genes in zebrafish. **(A-J)** Relative mRNA expression of Parkinson’s disease-associated genes in each group of zebrafish when they developed to 120 hpf. WT, wild-type ATP1A3 overexpression group; MUT, *ATP1A3* c.823G>C mutation overexpression group; WT+MUT, rescue group. NS, not significant, ^*^p < 0.05, ^**^p < 0.01, ^***^p < 0.001, and ^****^p < 0.0001.

Compared with the control group, the mRNA levels of *syn2b*, *pink1*, *sncgb*, and *sncb* were increased in the WT and MUT groups, while the mRNA level of *gba* was decreased, and the expression of these genes was partially restored in the WT + MUT group (Figures 6A,C,G,H,J). The mRNA levels of *gba* and *pink1* in the WT + MUT group were increased compared with the control group, but *syn2b*, *sncgb*, and *sncb* in the WT + MUT group were not significantly different from those in the control group (Figures 6A,C,G,H,J). The mRNA level of *dj1* in the WT group was higher than that in the control group, while the mRNA level of *dj1* in the MUT group was lower than that of the control and WT groups, and the mRNA level of *dj1* in the WT + MUT group was higher than that of the MUT group, and its expression level was similar to that of the control group (Figure 6E). On the contrary, the mRNA level of *polg* in the WT group was lower than that of the control group, while the mRNA level of *polg* in the MUT group was higher than that of the control and WT groups, and the mRNA level of *polg* in the WT + MUT group was decreased compared with that of the MUT group, and its expression level was close to that of the control group (Figure 6F). In addition, the mRNA level of *sncga* in the MUT group was higher than that in the control group, but there was no significant difference in *sncga* between the control, WT, and WT + MUT groups (Figure 6I). Overexpression of wild-type and mutant *ATP1A3* did not significantly affect the mRNA levels of *parkin* and *lrrk2* genes in zebrafish (Figures 6B,D).

### Effects of *ATP1A3* gene on the apoptosis-associated genes in zebrafish

**Figure 7:**
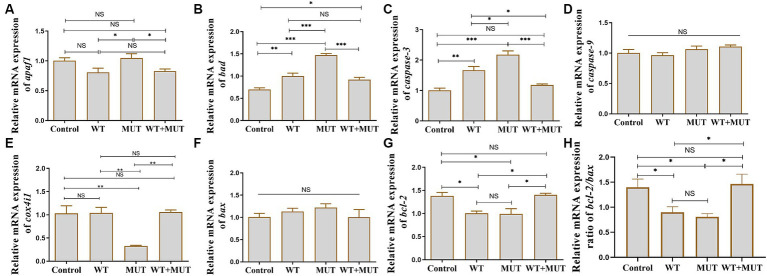
Effects of the *ATP1A3* gene on apoptosis-associated genes in zebrafish. **(A-H)** Relative mRNA expression of apoptosis-associated genes in each group of zebrafish when they developed to 120 hpf. WT, wild-type ATP1A3 overexpression group; MUT, *ATP1A3* c.823G>C mutation overexpression group; WT+MUT, rescue group. NS, not significant, ^*^p < 0.05, ^**^p < 0.01, and ^***^p < 0.001.

Compared with the control group, the mRNA levels of *bad* and *caspase-3* in the WT and MUT groups increased, while the mRNA levels of *bcl-2* and the ratio of *bcl-2/bax* decreased, and the expression of *bad*, *caspase-3*, *bcl-2* and the ratio of *bcl-2/bax* were partially restored in the WT + MUT group (Figures 7B,C,G,H). The mRNA level of *bad* in the WT + MUT group was higher than that in the control group, but the expression of *caspase-3* and *bcl-2* as well as the ratio of *bcl-2/bax* in the WT + MUT group were not significantly different from that in the control group (Figures 7B,C,G,H). The mRNA level of *apaf1* in the MUT group was higher than that in the WT and WT + MUT groups, but was not significantly different from that in the control group, and the mRNA level of *apaf1* was not significantly different between the control, WT, and WT + MUT groups (Figure 7A). Additionally, the mRNA level of *cox4i1* in the MUT group was significantly lower than that in the control, WT, and WT + MUT groups, whereas it was not significantly different between the control, WT, and WT + MUT groups (Figure 7E). Overexpression of wild-type and mutant *ATP1A3* did not significantly affect the mRNA levels of *caspase-9* and *bax* genes in zebrafish (Figures 7D,F).

## Discussion

*ATP1A3*-related neurologic disorders are rare, but over the past decade, they have garnered increased attention. With advancements in gene diagnosis technology, more than 60 different *ATP1A3* mutations have been considered to be related to neurological and psychiatric symptoms, and some cases demonstrated intermediate, overlapping, or atypical phenotypes (Nicita et al., 2016; Lax et al., 2021; Salles et al., 2021). This study, through *in vitro* experiments, has firstly identified that the *ATP1A3* p.Ala275Pro mutation leads to decreased levels of *ATP1A3* protein and Na^+^-K^+^-ATPase activity in cells. More importantly, we found that both overexpression of wild-type *ATP1A3* and the p.Ala275Pro mutant affect the growth and development (brain size, body length, and dopamine neuron development) as well as motor behavior of zebrafish. Moreover, through evaluating the gene expression profile in the zebrafish model, we also discovered differential regulation in transcription levels of dopamine signaling pathway-associated genes, PD-associated genes, and apoptosis signaling pathways. These results suggest that the *ATP1A3* p.Ala275Pro mutation may lead to dysfunction of *ATP1A3* protein and abnormal neurodevelopment in zebrafish. Additionally, increased expression of wild-type *ATP1A3* may also have harmful effects. Indeed, to date, functional studies of the *ATP1A3* p.Ala275Pro mutant have not been reported. Therefore, our research may provide new insights into the in-depth exploration of the potential pathogenic mechanisms of *ATP1A3*-related neurologic disorders.

Among *ATP1A3*-related neurologic disorders, RDP is characterized by sudden attacks of primarily bradykinesia and postural instability, often accompanied by marked medullary symptoms (de Carvalho et al., 2004; Hertz et al., 2023). The onset of RDP is usually triggered by physiological stimuli, with common triggers including excessive exercise, drinking, fever, and psychological stress (such as childbirth) (Brashear et al., 1993). The age of onset of RDP ranges from 9 months to 55 years. RDP attacks often occur within hours after the triggering event, and the disease can last from several hours to several weeks, with disease progression typically halts within 1 month, after which symptoms remain relatively stable in most patients (Brashear et al., 1993; Yu et al., 2022). In many patients with RDP, dystonia typically exhibits a rostro-caudal gradient in severity (symptom severity: face [bulbar] > arm > leg), and the symptoms of medulla oblongata and arm rarely improve after the initial attack (Heinzen et al., 2014). However, a cohort study found that the rostro-caudal severity gradient was uncommon among *ATP1A3* mutation carriers with at least mild symptoms of dystonia (7%). They observed that RDP symptoms most commonly started from focal and then progressed to generalized (51%) or multifocal (49%), with the arm being the most common site of first onset (41%) and the most severely affected (48%) (Haq et al., 2019). RDP was originally described as an autosomal dominant inheritance pattern, but recent studies have found that more than half of patients with RDP do not have a positive family history, and these cases may be caused by *de novo* mutation (Heinzen et al., 2014; Haq et al., 2019). Therefore, *ATP1A3* testing also needs to be considered in patients with dystonia with mid-adult onset, limb onset, and no family history.

In the spectrum of clinical phenotypes associated with *ATP1A3* mutations, AHC is one of the most prevalent. AHC is a rare and severe neurodevelopmental syndrome typically manifesting in infancy or early childhood (Hoei-Hansen et al., 2014). AHC is characterized by recurrent episodes of paroxysmal hemiparesis affecting one or both limbs, and these symptoms typically improve or resolve during sleep but recur upon waking (additional symptoms may include paroxysmal dystonic attacks, autonomic dysfunction, oculomotor abnormalities, seizure-like episodes, ataxia, parkinsonism, cognitive impairment, and various movement disorders) (Brashear et al., 1993; Perulli et al., 2022). Similar to RDP, hemiplegic attacks in AHC may be triggered by factors such as psychological stress, environmental stress, strenuous exercise, or illness. The frequency of these attacks can be high, occurring several times a day, week or month, with the duration of each attack ranging from a few minutes to even a few days (Kansagra et al., 2013). Statistically, approximately 75% of AHC cases are linked to mutations in *ATP1A3* encoding the α3 subunit of Na^+^/K^+^-ATPases, whereas most of these mutations are sporadic, caused by *de novo* mutations, with familial cases being rarely reported (Uchitel et al., 2021; Yang et al., 2021). Despite the growing number of pathogenic mutations identified in *ATP1A3*, the p.Asp801Asn, p.Glu815Lys, and p.Gly947Arg mutants remain the most frequently observed mutants causing AHC (Panagiotakaki et al., 2015; Cordani et al., 2021). Among these, the p.Asp801Asn mutant, which accounts for 30–43% of all cases, appears to be associated with a milder phenotype, causing a mild/moderate disease phenotype; the p.Glu815Lys mutant, which is associated with a more severe phenotype, leading to severe intellectual and dystonic deficits, as well as more frequent (or early-onset) seizures, with a poorer prognosis; and the p. Gly947Arg mutant has a better prognosis, with this mutation causing the latest onset of paroxysmal events compared to the other two mutations (Capuano et al., 2020).

The α subunit of Na^+^/K^+^-ATPase comprises three characteristic cytoplasmic structural domains (phosphorylation [P], nucleotide-binding [N], and actuator [A]), along with a transmembrane region consisting of 10 transmembrane helices (M1–M10) (Roenn et al., 2019). Most of the identified mutations associated with AHC occur in or near the transmembrane structural domain of the *ATP1A3* protein, while mutations related to RDP appear to be distributed more evenly throughout *ATP1A3*, indicating that mutations at specific sites within *ATP1A3* may specifically predispose individuals to AHC (Heinzen et al., 2012). In experiments involving cells transfected with various mutants associated with AHC and RDP, transfection of RDP-associated mutants resulted in a notable decrease in *ATP1A3* protein expression, whereas mutations leading to AHC did not significantly reduce *ATP1A3* protein expression (de Carvalho et al., 2004; Heinzen et al., 2012). Interestingly, Lazarov et al. (2020) observed significant differences in *ATP1A3* protein expression within the AHC mutant group, whereas no significant differences were noted in protein expression between the RDP mutant groups. In an animal model, heterozygous knockoutα3^+/KOI4^ mice (*ATP1A3* ^tm1/Ling^) exhibited a 60% reduction in α3 protein expression specifically in the hippocampus (Moseley et al., 2007). Additionally, in heterozygous knock-in mice (α3^+/D801Y^) carrying the D801Y mutation that can associated with AHC (Viollet et al., 2015) or RDP (Brashear et al., 1997; de Carvalho et al., 2004), the introduction of the D801Y mutation led to a significant decrease in α3 protein expression in the cortex, hippocampus, cerebellum, and in whole brain lysates of the mice (Holm et al., 2016). Moreover, Isaksen et al. also observed that α3^+/D801Y^ mice developed severe hypothermia-induced dystonia, and they found that α3^+/D801Y^ mice expressed approximately 80% of the α3 protein levels in the cerebellum compared to wild-type mice (Isaksen et al., 2017). These data suggest that certain mutations associated with AHC and RDP may impact the expression of the *ATP1A3* protein.

More importantly, sufficient Na^+^/K^+^-ATPase activity is crucial for maintaining normal function of the nervous system (Jiao et al., 2022). Calderon et al. (2011) found that by infusing ouabain into the cerebellum of mice to partially block the sodium pump, the initial motor symptoms in the model mice manifested as ataxia, and as the concentration of ouabain increased and the duration of exposure lengthened, the mice exhibited dystonia-like postures and eventually developed into generalized dystonia under high concentrations of ouabain. Ataxia has been progressively reported in *ATP1A3*-related neurologic disorders, and mild involvement of the cerebellar *ATP1A3* Na^+^/K^+^ pump is thought to be associated with *ATP1A3*-related ataxia (Schirinzi et al., 2018). However, dystonia may result from higher or prolonged inhibition of the *ATP1A3* Na^+^/K^+^ pump (Calderon et al., 2011; Schirinzi et al., 2018). Mutations of AHC and RDP lead to a decrease in Na^+^/K^+^-ATPase activity or pump current (de Carvalho et al., 2004; Weigand et al., 2014; Lazarov et al., 2020). Heterozygous *ATP1A3* ^tm1/Ling^ mice showed a 15% decrease in neuronal Na^+^/K^+^-ATPase activity compared to wild-type mice, and under chronic variable stress, neuronal Na^+^/K^+^-ATPase activity in *ATP1A3* ^tm1/Ling^ mice decreased by 33% compared with wild-type (Kirshenbaum et al., 2011). Additionally, studies have demonstrated that RDP mutants resulted in a significant decrease in the affinity of Na^+^/K^+^-ATPase for Na^+^, but its binding to K^+^ is not significantly impaired (Toustrup-Jensen and Vilsen, 2002; Blanco-Arias et al., 2009; Einholm et al., 2010). However, in AHC, the affinity of Na^+^/K^+^-ATPase for K^+^ may also be affected (Koenderink et al., 2003; Heinzen et al., 2014). In a study involving structural modeling of AHC mutations (I274N, D801N, D923Y) and RDP mutations (I274T, D801Y, D923N) affecting the same locations to analyze their impact on the structure of Na^+^/K^+^-ATPases α3, it was found that the AHC mutants significantly altered K^+^ conductance within the K^+^ pore, while RDP mutants had a comparatively minor effect on K^+^ passage (Kirshenbaum et al., 2013). This may explain the differences of the phenotype between AHC and RDP. In the family we studied, we identified a newly reported mutation site, p.Ala275Pro, which is adjacent to previously reported mutants such as p.Ile274Asn, p.Ile274Thr, and p.Glu277Lys. Structural modeling of the p.Ile274Asn mutation revealed that the Ile274Asn substitution resulted in Glu776 losing its interaction with K^+^, and the side-chain contact between Δ272 and Δ274 at the cytoplasmic terminus of the K^+^ pore was lost in the Ile274Asn mutant as compared to the wild-type protein (Kirshenbaum et al., 2013). These alterations could potentially disrupt the normal function of the proteins, leading to a reduction in ATPase activity (Weigand et al., 2014). Previous studies have also indicated that these three mutations can result in diminished Na^+^/K^+^-ATPase activity or reduced *ATP1A3* protein expression, thereby contributing to the development of AHC or RDP (de Carvalho et al., 2004; Weigand et al., 2014). However, in this study, the *ATP1A3* p.Ala275Pro mutation not only down-regulated *ATP1A3* protein expression in the cells compared to the wild-type group but also reduced Na^+^/K^+^-ATPase activity after the *ATP1A3* mutation compared to the wild-type group. Therefore, we hypothesize that the *ATP1A3* p.Ala275Pro mutation causing RDP and AHC in a mother and daughter pair in this family, respectively, is related to the fact that both *ATP1A3* protein levels as well as ATPases activity were affected by the mutation.

Most of the currently reported *ATP1A3* mutations are either associated with AHC or RDP, but it is possible that the same *ATP1A3* pathogenic mutations may lead to different phenotypes (Roubergue et al., 2013; Boelman et al., 2014). In rare instances, clinical features of AHC and RDP can overlap, and some patients may develop shared characteristics of AHC and RDP as the disease progresses (Termsarasab et al., 2015; Pereira et al., 2016; Tran et al., 2020). Common clinical features of AHC and RDP include asymmetric, predominantly dystonic movement disorders (Rosewich et al., 2014). Abnormalities in striatal dopaminergic neurotransmission have been considered part of the pathophysiology of dystonia (Lohmann and Klein, 2013; Di Fonzo et al., 2019). Patients with RDP often present with PD symptoms, and the degeneration and loss of nigrostriatal dopamine neurons causing dopamine deficiency in the striatum is also neuropathological features of PD (Wang et al., 2023). The decreased activity of Na^+^/K^+^-ATPase can affect the uptake and release of neurotransmitters such as dopamine, neuronal activity, and animal behavior. Therefore, dysregulation of the expression of genes associated with the dopamine signaling pathway may play a significant role in the pathogenesis of AHC and RDP. Interestingly, findings from studies on the effects of *ATP1A3* mutations on dopamine and its metabolites appear to be inconsistent. A previous study demonstrated a reduction in the level of homovanillic acid, a dopamine metabolite, in the cerebrospinal fluid of patients with RDP (Brashear et al., 1998). However, in heterozygous *ATP1A3* ^tm1/Ling^ mice, high-performance liquid chromatography (HPLC) analysis revealed no significant difference in dopamine and its metabolite levels in the striatum between stressed heterozygous mice and wild-type mice; the vertical activity of stressed heterozygous mice showed a negative correlation with the levels of dopamine and its metabolites, but this relationship was not observed in stressed wild-type mice (DeAndrade et al., 2011). In addition, Ikeda et al. (2017) reported that *ATP1A3* knockout homozygous mice (*ATP1A3*^−/−^) exhibited higher expression levels of monoamine neurotransmitters, particularly dopamine and norepinephrine, in the brain compared to wild-type mice. In a mice model of the RDP phenotype resulting from simultaneous perfusion of the mice’s striatum and cerebellum using the ATP1α3-blocker ouabain and exposure of the mice to mild exercise stress, HPLC analysis revealed a significant decrease in striatal dopamine content and an increase in dopamine conversion rate in RDP mice compared to controls, and qPCR analysis demonstrated significant up-regulation of dopamine receptor *Drd4* mRNA expression(a possible compensatory mechanism); exercise stimulation nearly restored dopamine concentration in RDP mice to control levels, while *Drd4* mRNA expression was down-regulated (Rauschenberger et al., 2020). In contrast to RDP mice, exercise stress did not influence the levels of dopamine and its metabolites in the striatum of mice perfused with sodium chloride, indicating that the striatum was particularly susceptible to stress after inhibition of Na^+^/K^+^-ATPase activity by ouabain (Rauschenberger et al., 2020).

The brain requires significant Na^+^/K^+^-ATPase activity, as this pump consumes approximately 50% of the energy in the CNS (Shrivastava et al., 2020). Moreover, Na^+^/K^+^-ATPase is crucial for maintaining Na^+^ and K^+^ homeostasis and is involved in the regulation of apoptosis, which is pivotal in various neurological disorders (Panayiotidis et al., 2006). In a rat model of cerebral ischemia/reperfusion injury, Na^+^/K^+^-ATPase activity was found to be reduced in the model group compared to the sham-operated group, and Na^+^/K^+^-ATPase activity exhibited a significant positive correlation with the *bcl-2/bax* ratio and a significant negative correlation with the score of neurological deficits as well as the percentage of apoptotic neurons (apoptotic index) (Huang et al., 2015). This aligns with findings that inhibiting Na^+^/K^+^-ATPase activity with ouabain can rapidly induce apoptosis in cell lines (de Carvalho et al., 2004; Lazarov et al., 2020). In fibroblasts obtained from patients with AHC, there was a significant increase in active cathepsin B levels, leading to a more pronounced activation of apoptosis (Di Michele et al., 2013). Our findings are consistent with this. In our zebrafish model, we overexpressed wild-type *ATP1A3* and the *ATP1A3* c.823G > C mutation, we observed that both wild-type and mutant *ATP1A3* overexpression led to increased mRNA levels of the pro-apoptotic genes *bad* and *caspase-3*, along with decreased mRNA levels of *bcl-2* and a reduced ratio of *bcl-2/bax*. This suggests that abnormalities in both wild-type and mutant *ATP1A3* expression may contribute to aberrant apoptosis. Na^+^/K^+^-ATPases is a prominent membrane protein in the brain, and defects caused by missense mutations in transmembrane proteins likely arise from misfolding during biosynthesis in the endoplasmic reticulum, triggering the unfolded protein response (Arystarkhova et al., 2021). The unfolded protein response initially serves as a defense mechanism, but prolonged activation can trigger apoptosis, which aligns with the apoptosis-related changes we observed in zebrafish. Although these studies provide evidence that *ATP1A3* mutations may disrupt apoptosis regulation, the precise underlying mechanism requires further investigation.

The homology between the zebrafish and human genomes is remarkably high, reaching up to 87%, and the formation of zebrafish dopamine neurons begins at ~24 hpf and are fully developed at at ~48 hpf (Du et al., 2016). Deficiency in Na^+^/K^+^-ATPases α3 in zebrafish results in brain ventricle dilation and alters their response to tactile stimuli, and zebrafish exhibit abnormal motility (Doganli et al., 2013). This suggests that Na^+^/K^+^-ATPases α3 may play a role in regulating brain volume and controlling motility in zebrafish. In our study, we investigated the effects of wild-type *ATP1A3* and the p.Ala275Pro mutant on the growth, development, and movement behaviors of zebrafish. We observed that compared to the control group, zebrafish overexpressing wild-type *ATP1A3* and those overexpressing the mutant *ATP1A3* exhibited smaller brains and body lengths, reduced fluorescence intensity of dopamine neurons, diminished escape responses, and decreased swimming distances and average movement speeds. These effects were partially improved in the rescue group. Additionally, overexpression of both wild-type and mutant *ATP1A3* influenced the expression of genes associated with the dopamine signaling pathway and Parkinson’s disease in zebrafish at the transcriptional level. Moreover, they dysregulated the mRNA expression of genes related to apoptosis. Therefore, we speculate that abnormal expression of wild-type and mutant *ATP1A3* dysregulates neurodevelopment-related genes and apoptosis-related genes in zebrafish, at least at the transcriptional level, resulting in impairment on zebrafish growth, development, and movement behavior. It has been suggested that *ATP1A3* mutants can cause protein misfolding and impaired trafficking of α3 (Arystarkhova et al., 2019, 2021). Additionally, the total amount of α-subunits of Na^+^/K^+^-ATPases is constant, and the introduction of wild-type exogenous α3 can compete with the endogenous α1 protein, thereby affecting protein expression (Arystarkhova et al., 2019). This may explain why overexpression of both wild-type and mutant *ATP1A3* is detrimental in our zebrafish model.

In conclusion, the spectrum of clinical and genetic mutations of *ATP1A3* mutations is broadening, with different mutations potentially leading to distinct clinical phenotypes through varying mechanisms. Here, we demonstrate that the p.Ala275Pro mutant reduces *ATP1A3* protein expression and ATPase activity in cells. Moreover, abnormalities in both wild-type and mutant *ATP1A3* expression are detrimental. Our zebrafish model provides further evidence supporting the pathogenic role of the *ATP1A3* p.Ala275Pro mutant in neurodevelopment. These studies may serve as a reference for future investigations into the mechanisms underlying AHC and RDP.

## Data availability statement

The original contributions presented in the study are included in the article/Supplementary material. Further inquiries can be directed to the corresponding author.

## Ethics statement

The studies involving humans were approved by the Ethics Committee of Fujian Provincial Hospital. The studies were conducted in accordance with the local legislation and institutional requirements. Written informed consent for participation in this study was provided by the participants’ legal guardians/next of kin. The manuscript presents research on animals that do not require ethical approval for their study.

## Author contributions

D-dR: Writing – original draft. JZ: Data curation, Writing – original draft. L-sL: Data curation, Writing – original draft. M-dJ: Data curation, Writing – original draft. R-lW: Writing – original draft. J-hZ: Writing – original draft. LZ: Writing – original draft. M-zG: Writing – original draft. QC: Writing – original draft. H-pY: Writing – original draft. WW: Writing – review & editing. Y-fL: Writing – original draft. HL: Writing – review & editing. FL: Writing – review & editing. J-wL: Writing – review & editing. X-fL: Writing – review & editing.
