# Supplementary material for: In vitro study of ATP1A3 p.Ala275Pro mutant causing alternating hemiplegia of childhood and rapid-onset dystonia-parkinsonism
Source: Front Neurosci. 2024 Jul 31;18:1415576. doi: 10.3389/fnins.2024.1415576 (PMC11322359; doi:10.3389/fnins.2024.1415576)
Supplement: Supplementary file 1 [file Data_Sheet_1.pdf]

Case presentation

In previous clinical work, a family was identified in which genetic analysis revealed that both the proband and her mother carried a heterozygous mutation c.823G>C (p. Ala275Pro) in exon 8 of *ATP1A3* (NM_152296.4). However, the proband and her mother exhibited different clinical phenotypes of AHC and RDP, respectively (12). The proband in this family is a girl aged 2 years and 11 months. Her medical history includes experiencing fever at the age of 2 years and 5 months, lasting for one week, after which the symptoms resolved spontaneously. However, 3 weeks later, she developed paroxysmal hemiplegia characterized by episodes of limb weakness while awake, which typically improved during sleep. These episodes occurred 1-2 times per month, with involvement of both limbs alternately showing weakness. In addition to hemiplegic attacks, the proband also occasionally experienced ocular motility disorders or dysarthria, which could occur concurrently or independently of the hemiplegic episodes. Notably, there were no other neurological abnormalities such as ataxia, dysphagia, or choreoathetosis, nor were there symptoms indicative of autonomic imbalance like hyperhidrosis or low blood pressure. Various diagnostic tests including biochemical examinations, autoimmune encephalitis-related antibody testing, brain magnetic resonance imaging (MRI), and electroencephalography (EEG)did not reveal any significant abnormalities. Ultimately, based on the results of genetic analysis, the proband was diagnosed with AHC. When the proband's mother was 22 years old, she developed several neurological symptoms including left limb weakness, difficulty lifting, involuntary shaking of the left upper limb, inability to grasp with the left hand, left foot varus (inward deviation), and bradykinesia (slowness of movement). These symptoms manifested about one month after childbirth. Over the subsequent month, the symptoms stabilized with occasional periods of improvement. Despite stabilization, she was left with lasting sequelae such as clumsiness in the left limb, involuntary shaking of the left upper limb, and a specific posture of left upper limb extension when the right upper limb was used. Diagnostic investigations revealed mild abnormalities on EEG, without spike-sharp wave discharges. Brain MRI showed slight cerebellar atrophy, while electromyography indicated neurogenic damage characterized by slowed conduction velocities in the left ulnar and radial nerves. Based on comprehensive genetic analysis, the proband's mother was diagnosed with RDP.
